# Inflammatory Bowel Disease: Crosstalk between Histamine, Immunity, and Disease

**DOI:** 10.3390/ijms24129937

**Published:** 2023-06-09

**Authors:** Kristina A. Dvornikova, Olga N. Platonova, Elena Y. Bystrova

**Affiliations:** I.P. Pavlov Institute of Physiology RAS, St. Petersburg 199034, Russia; 691442@gmail.com (K.A.D.);

**Keywords:** histamine, histamine receptors, HDC, HNMT, DAO, intestine, inflammatory bowel disease, ulcerative colitis, Crohn’s disease, inflammation, immunity, mast cells, polymorphism

## Abstract

Inflammatory bowel disease (IBD) is increasingly recognized as a serious, worldwide public health concern. It is generally acknowledged that a variety of factors play a role in the pathogenesis of this group of chronic inflammatory diseases. The diversity of molecular actors involved in IBD does not allow us to fully assess the causal relationships existing in such interactions. Given the high immunomodulatory activity of histamine and the complex immune-mediated nature of inflammatory bowel disease, the role of histamine and its receptors in the gut may be significant. This paper has been prepared to provide a schematic of the most important and possible molecular signaling pathways related to histamine and its receptors and to assess their relevance for the development of therapeutic approaches.

## 1. Introduction

Inflammatory bowel disease (IBD) is a group of chronic, immune-mediated disorders of the gastrointestinal tract (GI) that includes ulcerative colitis (UC) and Crohn’s disease (CD), with complex pathophysiology and pathogenesis [1]. A dysregulated innate and adaptive immune response may be the result of a complex interaction between genetic predisposition, environmental factors, epithelial barrier defects, and altered gut microbiota in IBD [2,3]. In UC, Th2 activity increases due to the release of interleukin-4 (IL-4), IL-5, IL-13, and TNF-α, while in CD, Th1 and Th17 activity increases due to the production of IFN-γ and IL-2, IL-12, IL-18, and interleukin-23 (IL-23), which are produced by antigen-presenting cells (APCs) and macrophages [4]. In recent years, a number of works has also focused on the study of correlations between IBD-associated genes [5], single nucleotide polymorphism, dysbiosis [6,7], and the severity of the disease. However, these issues are still under consideration.

Histamine has been a subject of research since the 1910s, and since then, our knowledge of its functions and how it affects both physiological and pathophysiological processes has expanded significantly [8]. In humans, histamine is found in almost all body tissues, where it is mainly stored in the granules of mast cells and basophils. In addition, inflammatory cells, such as neutrophils and macrophages, can operate as additional sources of histamine release in the body [9]. The important external sources such as dietary intake and microbiota secretion shall also be taken into account when considering the potential role of this important amine in gastrointestinal homeostasis [10,11].

A growing body of the literature has investigated the role of histamine and its receptors (HRs); however, their effect on the development of IBD is still far from being understood [12,13]. All HRs except H3R were found to be expressed in the human gut [14]. Proinflammatory effects for H1R and H4R [15,16] and anti-inflammatory effects for H2R [17,18] have been demonstrated in IBD. Moreover, the influence of histamine receptors on the development of Th1/Th2/Th17 and regulatory T-cells (Tregs) from CD4^+^ progenitor cells, which may be crucial for the progression of inflammation, has been demonstrated by several studies [19,20,21].

There has been a paucity of the literature on a comprehensive analysis of HRs-associated molecular pathways in IBD. The diversity and interaction of numerous histamine sources, signaling pathways, and molecules have not been well explored to unravel the true mechanisms underlying the HR’s regulatory role in the gut. The main functions of histamine receptors in the healthy and inflamed intestines, as well as their synergies, are outlined in this review. We also evaluated the experimental data from the literature of the last few years, on the basis of which we present in this review a schematic of the main and potential molecular signaling pathways associated with histamine receptors in IBD.

## 2. General Information on Histamine

### 2.1. Structure, Biosynthesis, and Degradation of Histamine

Since the discovery of histamine (2-[3H-imidazol-4-yl]ethanamine) more than a century ago, our understanding of its roles in both physiological and pathophysiological processes has significantly increased [7,22].

L-histidine decarboxylase (HDC, EC 4.1.1.22) employs the enzymatic decarboxylation of the amino acid L-histidine to produce histamine [8,23]. Histamine is sorted and packed into the secretory granules of mast cells, basophils, and histaminergic neurons by the Golgi apparatus, and is then released after cell sensitization and degranulation [24]. Cell degranulation and histamine release occur when a specific antigen binds to the FcepsilonRI (FcεRI) receptor on the surface of mast cells and basophils, or in response to nonimmune stimuli [25]. Crosslinking of the immunoglobulin E (IgE)/FcεRI complex with antigen causes the activation of tyrosine kinases via a common signaling pathway, Fyn-Lyn-Syk, followed by phospholipase C (PLC) activation. PLC hydrolyzes the membrane phosphatidylinositol 4,5-diphosphate (PIP2) to produce diacylglycerol (DAG) and inositol-1,4,5-triphosphate (IP3). These second messengers activate protein kinase C (PKC) and increase cytosolic Ca^2+^, triggering the degranulation and synthesis of proinflammatory lipid mediators, as well as the production of cytokines and chemokines.

Histamine can also be released in other ways (e.g., due to tissue injury). As a result, a number of cytokines, including interleukin-3 (IL-3), interleukin-18 (IL-18), interleukin-33 (IL-33), granulocyte-macrophage colony-stimulating factor (GM-CSF), and stem cell factor (SCF), stimulate the production of histamine. In addition, exogenous compounds, inflammatory mediators (e.g., chemokines, prostaglandins), β-defensins, and neuropeptide substance P (SP) can all induce histamine synthesis.

Diamine oxidase (DAO, EC 1.4.3.22) is the principal enzyme responsible for histamine catabolism [26]. The oxidative deamination reaction is initiated by DAO, which converts histamine to imidazole acetaldehyde. Histamine-N-methyltransferase (HNMT, EC 2.1.1.8) is another enzyme involved in histamine degradation, which transfers a methyl group from S-adenosyl-L-methionine (SAM) to histamine, producing N-methylhistamine (NMH) that is further converted to N-methylimidazole acetic acid [27].

The rate of histamine degradation differs among individuals and can be influenced by various factors such as genetics, diet, and underlying medical conditions. Impaired histamine degradation can lead to the development of histamine intolerance (HIT), a nonimmune reaction characterized by the accumulation of histamine in the body due to decreased histamine breakdown capacity [28]. Additionally, some drugs and chemicals can affect histamine synthesis and release, often resulting in allergic and inflammatory reactions.

### 2.2. Function of Histamine in the Body

Histamine is involved in a wide range of physiological and pathological conditions, including neurotransmission, immunomodulation, cell proliferation and differentiation, hematopoiesis, carcinogenesis, embryonic development, regeneration, and regulation of gastrointestinal (GI) and cardiovascular system functions [29].

It should be noted that our understanding of histamine’s role in the GI is still evolving. Modulation of intestinal smooth muscle contraction, promotion of hydrochloric acid secretion in the stomach, and vascular vasodilation, enabling the maintenance of homeostasis throughout the tract, are recognized as important functions of histamine in the GI. Furthermore, it is clear that histamine mediates pathogenic conditions, in particular those of the digestive system, by directly or indirectly modulating the immune response [30,31]. In light of the underlying immunological crossover pathways between intestinal inflammation and the histamine signaling cascade, it is also important to consider the potential function of histamine in chronic inflammatory diseases. The third part of this review will explore this issue in depth.

### 2.3. Sources of Histamine

Histamine originates from an array of sources and can be endogenous (i.e., produced by the body itself) or exogenous (i.e., obtained from external sources) (Table 1).

Histamine can be found in a variety of foods, especially those that have been aged, fermented, or otherwise processed. In addition to histamine, these foodstuffs contain cadaverine (pentane-1,5-diamine) and putrescine (1,4-diaminobutane). Cadaverine and putrescine compete with histamine for DAO binding sites, inhibiting the breakdown of histamine DAO, resulting in the accumulation of histamine in the body and the development of HIT [35]. High concentrations of histamine are found in microbial fermentation products that require favorable conditions for the growth of decarboxylase-positive bacteria and decarboxylase activity.

The gut microbiome contains many Gram-positive and Gram-negative bacteria that encode HDC and release the biogenic amine histamine [10]. It is known that the bacterial production of amino acid decarboxylase is increased in an acidic environment, resulting in a local increase in pH around the bacteria and protection against a chloride-rich acidic environment. Furthermore, the presence of fermentable carbohydrates and oxygen, the redox potential of the medium, the temperature, and the concentration of sodium chloride (NaCl) influence the expression and activity of decarboxylases in bacteria [36]. However, our understanding of the immunoregulatory activity of histamine in the GI has been insufficient to provide a comprehensive explanation of the molecular biology of the histamine system. Nonetheless, recent findings enable us to assess its possible opportunities and risks associated with intestinal disorders.

### 2.4. Histamine Receptors

The pleiotropic effects of histamine are mediated by four HRs and can be beneficial or harmful depending on the context and balance between histamine and its regulatory mechanisms (Table 2) [37].

Histamine is an endogenous HR ligand with a moderate to high affinity for HRs: the affinity for H1R and H2R is more than 1000-fold lower than the affinity for H3R and H4R, although this lack of selectivity is a significant disadvantage [38].

Each subtype of HRs activates different signaling pathways and immunological activity. The effects of histamine can be both pro- and anti-inflammatory, depending on which HR is activated and the type and location of cells expressing that HR.

**Table 2:** Histamine receptors.

Receptor Subtype	G Protein Coupling	Expression	Molecular Mass (kDa)	Signal Transduction	References
H1	Gαq	Smooth muscle cells of the respiratory and cardiovascular systems, endothelial cells, enterocytes, monocytes, neutrophils, T- and B-lymphocytes	56	PLC and PKC activation, cytosolic Ca^2+^ increase, protein phosphorylation and transcription of nuclear factor κB (NF-κB), nuclear factor of activated T-cells (NFAT), cyclic adenosine monophosphate (cAMP), response element binding protein (CREB), and activator protein 1 (AP-1)	[30,39]
H2	Gαs	Heart tissue, enterocytes, brain cells, smooth muscle cells, T- and B-cells, and DCs	40	Adenylyl cyclase (AC) activation, increases cAMP, and activates protein kinase A (PKA)	[40]
H3	Gi/o	Histaminergic neurons, monocytes, eosinophils	48	Inhibits cAMP synthesis, causes Ca^2+^ accumulation, and activates the mitogen-activated protein kinase (MAPK) pathway	[41,42]
H4	Gi/o	Neutrophils, eosinophils, T cells, bone marrow cells, peripheral hematopoietic cells, thymus, lungs, small and large intestines, and heart	44	Inhibits cAMP synthesis, causes Ca^2+^ accumulation, and activates the mitogen-activated protein kinase (MAPK) pathway	[13]

Thus, the interaction with different HRs enables a variety of histamine effects in the body and also allows for the multidirectional (selective) regulation of various physiological and pathophysiological processes; in particular, inflammation.

## 3. The Role of Histamine in Intestine

### 3.1. Histamine and Intestine Inflammation

Significant mediators of inflammation include histamine. Consequently, histamine-mediated inflammation may help to establish a chronic inflammatory response, inter alia, in the gut [36]. The mechanisms underlying histamine’s pathological effects in the inflamed intestine, however, remain poorly understood as of this writing. Nevertheless, the information that is currently available enables us to reflect on some of the prime histamine-induced reactions that take place as well as to hypothesize that histamine may have an impact on the progression of inflammation.

Allergies, inflammation, altered DAO and HNMT activity, food intake, and microbiota are just a few factors that can affect gut histamine levels [36]. Therefore, an increase in histamine levels in the body can affect intestinal homeostasis, the occurrence of HIT, and the development of food allergies or other histamine-related conditions, all of which impact the functional state of the gut. It should be noted that not enough research has been conducted on how histamine affects the intestinal immune response to food antigens [43]. However, histamine and HRs may be directly or indirectly involved in the mechanisms of tolerance and sensitization to food antigens. Additionally, histamine has the capacity to sensitize nerve endings, modulate pain signals, and is crucial for the emergence of the visceral hypersensitivity (VH) reaction in the intestine [44]. At the same time, H1R and H4R antagonists are able to, in a dose-dependent manner, lessen post-inflammatory VH.

Histamine and HRs are considered to be among the most important components of the signaling pathway in the context of influencing the development of IBD [12,13]. Given the high immunomodulatory activity of histamine and the complex immune-mediated nature of inflammatory bowel disease, the role of intestinal histamine in IBD may be significant.

### 3.2. General Description of Inflammatory Bowel Disease

It has been demonstrated that in IBD, an imbalance between Th17 and Tregs, which differentiate from CD4^+^ T-cells, promotes tissue inflammation and suppresses autoimmune reactions [20]. Th17 is a source of several proinflammatory cytokines: IL-17A, IL-17F, interleukin-22 (IL-22), interleukin-26 (IL-26), and the chemokine CCL20. At the same time, Th17 activity is regulated by IL-23 (activation) and IL-10 (inhibition). By releasing IL-10, monocytic cells and Tregs can suppress Th17 cell differentiation through TGF-β signaling and FoxP3 expression, reducing inflammation in IBD.

In addition to developing defects in the regulation of innate and adaptive immune responses, inflammation in IBD is known to cause intestinal barrier dysfunction and gut dysbiosis [7]. The intestinal barrier comprising IECs, enteroendocrine, innate immune cells, and intraepithelial lymphocytes (IELs) is the first physical and chemical barrier that intestinal bacteria, pathogens, and food antigens come into contact with [45]. It has been found that increased IL-17A, IFN-γ, and TNF-α production by IELs can cause inflammation and support IBD development [46]. In maintaining intestinal homeostasis and mucosal integrity, a significant role is assigned to TLRs. They identify endogenous chemicals or microorganisms with pathogen-associated molecular patterns (PAMPs), which activate the innate immune response [47]. However, increased TLRs signaling can directly or indirectly affect the development of IBD. For example, the expression of the TLR2, TLR4, TLR8, and TLR9 genes is upregulated in active UC, while the expression of the TLR5 gene is upregulated in active UC and downregulated in quiescent UC [48]. As a result, increased expression of one or another TLRs causes a variety of outcomes, ranging from mucous membrane inflammation to suppression of the immune response in the gut.

Due to functional disruptions in host–microbiota interactions caused by IBD-associated microbial dysbiosis, disease progression is modulated [6]. Microbiota dysbiosis in IBD is known to be characterized by a depletion of Firmicutes and Bacteroidetes and an increase in Proteobacteria and Actinobacteria. Ongoing studies on metagenomic composition also allow for the prediction of the altered metabolic pathways of the microbial community characteristics of IBD [49]. However, it is still unclear whether dysbiosis is a cause or a consequence of IBD.

A genetically predisposed person is also at risk of developing and progressing IBD [5]. At present, 200 IBD-associated loci have been identified and include, in particular, the following genes: NOD2, ATG16L1, IRGM, LRRK2, PTPN2, IL23R, Il10, Il10RA, Il10RB, CDH1, and HNF4α. Based on their function in the inflammatory pathway, these genes can be divided into three groups: those that recognize pathogens, those that facilitate innate immunity cells to eliminate them, and those that prevent pathogens from crossing the intestinal barrier. Discovering these associations would greatly improve the diagnosis of IBD. However, there are still no precise data reflecting the possible mutual influence of these genes and their SNPs on histamine and histamine pathways in IBD. To prevent the identification of erroneous positive associations with IBD, it is crucial in genetic screening to consider other (clinical, histological) data as well as other disease-related factors.

### 3.3. The Role of Histamine in IBD

#### 3.3.1. Mast Cells and IBD

Mast cells are present throughout the digestive tract and play an important role in the pathogenesis of IBD [50]. Early studies showed that mast cells of the resected colon with active CD and UC released more histamine than normal colonic mast cells [51,52]. A marked increase in the number of mast cells was also observed in the mucosa of the ileum and colon in IBD, which was accompanied with high levels of TNF-α, interleukin-16 (IL-16), and SP expression [53]. Mast cells play an important role in the development of dextran sulfate sodium (DSS)-induced colitis [54]. Thus, DSS colitis was induced in Ws/Ws rats with mast cell deficiency. It is known that mast cells are closely associated with the development of edema and tissue hyperemia. As a result, histamine levels in the colonic mucosa were significantly reduced compared to the controls. This demonstrates the role of mast cells’ activation in the pathogenesis of DSS-induced colitis. Another study has shown the role of mast cells and neuropeptides (SP, somatostatin (SS), and vasoactive intestinal peptide (VIP)) in DSS colitis [55]. It has been established that in the acute stage of DSS colitis in rats, the concentration of histamine in the colon tissue is significantly lower than in the control group. At the same time, the level of histamine in the whole blood was higher compared to the controls, which indicates the possibility for mast cells to directly participate in the process of mucosal damage by releasing histamine. In addition, it is reported that in DSS colitis, the increase in the histamine level in the whole blood and mast cells’ quantity in the distal colon tissue takes place before the increase in the SP levels. This suggests that activated mast cells promote SP release from adjacent nerve endings via the release of mediators, and thus, in turn, may be involved in the progression of inflammation and exacerbation of DSS colitis.

##### Mast Cells and Adaptive Immunity

In the lamina propria of IBD patients, more mast cells were observed throughout the tissue and more macrophages were noticed in the lower lamina propria. The release of proinflammatory mediators from accumulated mast cells may explain this recruitment of macrophages to the lamina propria. In addition, CD-related mucosa of the duodenum, colon, and ileum showed mast cell degranulation. The release of a variety of mediators, such as histamine, proteases, chemokines, and cytokines, is known to occur as a result of IgE/FcεRI-mediated mast cell degranulation, a traditional mechanism for mast cell activation [56]. Consequently, inflammatory cells are recruited, barrier functions are impaired, and as a result, intestinal permeability increases, which contributes to an increased influx of intraluminal antigens that activate mast cells or CD4^+^ T-cells, further stimulating inflammation. All of this combined causes further damage to the gut. However, at the same time, IL-2 and IL-10 released by mast cells inhibit excessive immune responses, thereby reducing the spread of damage [56]. In fact, a reduced number of IL-10-positive lymphocytes was found in the intestinal mucosa of DSS mice with histamine deficiency, HDC knockout, and altered fecal bacterial biota [57]. This suggests that histamine plays a role in the pathophysiology of inflammation in the colon by enhancing local IL-10 production and stimulating a local transition to a Th2 response.

##### Mast Cells and Innate Immunity

Peptidoglycan (PGN), a component of the bacterial cell wall, is a prime example of a conserved PAMPs that does not directly affect the barrier function of human IECs monolayers. Recent research has reported quantitative evidence that T84 IECs line transports PGN to activate mast cells and elevate the permeability of the T84 monolayer. The study also demonstrates that the human mast cell line HMC-1 expresses TLR2 and NOD2, which are crucial for PGN-impaired gut barrier function [58]. Accordingly, mast cell signaling in IBD is both pathological and potentially protective, depending on exposure to various receptors, intestinal cross-signaling outcomes, and other factors. However, the precise mechanisms of this signaling in human IBD have not been determined.

Mast cells have been shown to express NOD2 in an IFN-γ-dependent manner [59]. It has also been established that mast cells regulate the migration and activation of leukocytes and monocytes, primarily through NOD2 in IBD (particularly in CD). These results support the fact that mast cells can play a critical role in host defense.

##### Mast Cells, Enzymes, and Metabolites

Mucosal mast cells produce tryptase, which contributes to the progression of IBD caused by intestinal fibrosis by activating the major fibroblast PAR-2/Akt/mTOR (protease-activated receptor-2 (PAR-2)/protein kinase B (PKB, Akt)/mammalian target of rapamycin (mTOR)) pathway [60]. Chronic intestinal inflammation and infection lead to intestinal fibrosis, which is its pathological end result. Tryptase modulates the differentiation of fibroblasts into myofibroblasts with a fibrotic phenotype mainly via the PAR-2/Akt/mTOR pathway. The release of histamine by mast cells is then modulated by tryptase. However, histamine can also promote fibroblast proliferation, which, in turn, can lead to fibrosis. Histamine, therefore, plays an initiating role in fibrosis and probably in IBD mediated by intestinal fibrosis.

It has been noted that NMH, the primary metabolite of mast cell histamine, is a promising predictor because it captures the clinical and endoscopic activity of IBD [61]. Specifically, the detection of elevated urinary NMH levels in active CD and UC was correlated with disease activity. This result was probably influenced by mediators released by histamine-containing cells. However, NMH is not currently used as an indicator to monitor IBD activity in humans due to circumstantial evidence that NMH is ineffective [62].

It is obvious that mast cells, the primary histamine producer, play a critical role in the onset of inflammation. Studies to date have shown proinflammatory rather than anti-inflammatory effects of histamine on the gut in IBD. However, due to the complex multifactorial crossover pathways of the immune system, with variable outcomes, it is extremely difficult to formulate the precise molecular mechanisms underlying these responses. Additional research is needed.

#### 3.3.2. HRs and IBD

It is known that the human intestine expresses all HRs, with the exception of H3R [14]. It is still debatable whether H3R is present in human intestinal tissues, so more research using H3R-sensitive techniques is necessary [63]. It has been reported that IBD alters the expression and functional activity of HRs [30].

##### Histamine Receptor 1

By activating the H1R, histamine triggers ion transport in the gut, which stops by inhibiting the H1R [64]. It has been observed that H1R primarily promotes and/or amplifies proinflammatory responses. Consequently, the inflammatory focus in the intestine is subject to the increased chemotaxis of eosinophils and neutrophils, facilitating the activation of signaling pathways that trigger the synthesis of proinflammatory cytokines [15]. Additionally, it has been shown that H1R and H4R signaling synergizes cAMP accumulation and MAPK activation to increase the expression of proinflammatory genes [16]. However, the role of H1R in the pathogenesis of IBD is not yet fully established and needs further investigation.

##### Histamine Receptor 2

Histamine can suppress proinflammatory responses caused by the bacterial ligands of TLR2, TLR4, TLR5, and TLR9 receptors via H2R [12]. In particular, histamine was reported to potently suppress TLR-induced cytokine secretion by healthy volunteer peripheral blood mononuclear cells (PBMCs), but not by the PBMCs of patients with UC and CD, whereas famotidine administration reversed this suppressive effect. At the same time, blocking H2R worsened the disease progression. Histamine suppressed the secretion of IFN-γ and TNF-α, and the gene expression of these cytokines was positively correlated with the expression of H4R and H2R in UC. Consequently, the influence of H2R signaling can suppress excessive TLR responses to bacteria in the gut. Histamine-secreting Lactobacillus rhamnosus has been reported to suppress IL-2, IL-4, IL-5, IL-12, TNF-α, and GM-CSF secretion indirectly via H2R in wild-type mice but not in H2R-deficient mice [65]. In another study, it was established that Lactobacillus reuteri activates H2R and has an anti-inflammatory effect in mice with TNBS colitis [66]. The ratio of proinflammatory IL-1β and IL-6 in the mucosa was decreased in mice treated with L. reuterie. As can be seen, H2R demonstrates anti-inflammatory effects on IBD. The exact mechanisms of microbiome-mediated immunomodulation involving H2R and histamine in the human gut remain unclear. Hopefully, future investigations will allow for the development of next-generation probiotics and microbial-derived drugs for IBD therapy.

##### Histamine Receptor 4

According to experimental models, histamine plays a proinflammatory role in IBD via H4R [67]. Unfortunately, because it is impossible to accurately replicate all disease factors, IBD models cannot completely reflect actual changes in gut homeostasis. Typically, chemically induced acute inflammation is used in colitis models to simulate acute inflammation, providing a general understanding of the molecular and signaling pathways involved in the inflammatory process. Thus, during acute DSS-induced colitis in mice, H4R activity caused the increased expression of IL-5, IL-6, IL-10, IL-17, and IFN-γ after restimulation by αCD3 antibodies in lymph node cells compared to the control. This shows the acute immune response of the intestinal mucosa. Delayed disease onset, milder symptoms, and histological abnormalities in the colon were observed in H4R knockout mice or pharmacological H4R inhibition. These mice also showed lower cytokine production. Therefore, H4R contributes to the proinflammatory immune response of the colon. According to other research, H4R appears to play a proinflammatory role. In particular, mast cell histamine and H4R have been shown to promote an environment that allows pathogenic neutrophils to infiltrate the colonic mucosa and exacerbate symptoms of experimental colitis [68]. It has also been found that in oxazolone (Ox) and DSS-induced colitis, H1R and H2R knockout mice elicit a competent response via H4R, exacerbating the immunopathology of the disease [69]. Regarding that, H4R knockout mice had lower levels of symptoms, IL-6 infiltration, CXCL1, CXCL2, and mucosal neutrophils than wild-type mice. However, another study reported that the lack of H4R expression inversely worsened symptoms of 2,4,6-trinitrobenzenesulfonic acid (TNBS)-induced acute colitis in mice [70]. In comparison to the controls, mice with TNBS colitis and H4R knockout expressed higher levels of chemokines CXCL1 (KC) and CXCL2 (MIP2), IL-6, and the neutrophil enzyme myeloperoxidase (MPO). As a result, the findings point to a potential anti-inflammatory function of H4R that may have helped the inflammation resolve successfully. These findings, however, conflict with those of other research. However, it is crucial to remember that this study only used a small sample and a different model of chemically induced colitis, so the findings cannot be categorically interpreted.

#### 3.3.3. HRs Signaling Pathways in Context of IBD

**Figure 1:**
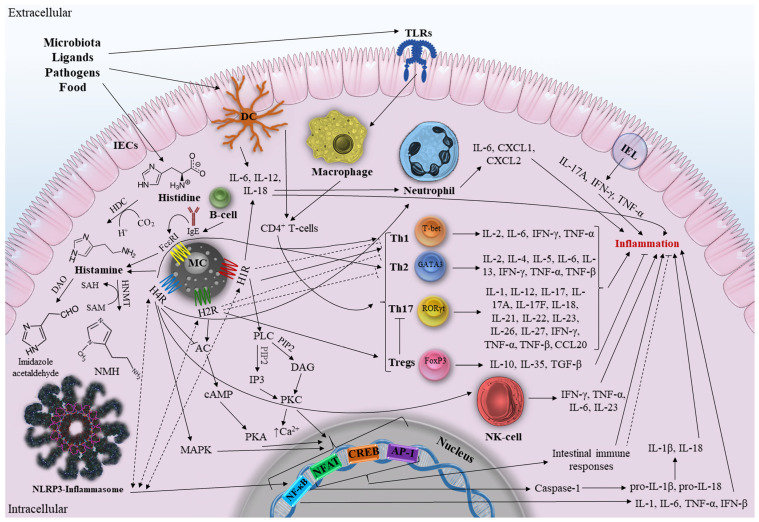
Major and possible molecular pathways associated with HRs in IBD. The dotted lines indicate the suggested paths. SAH, S-adenosylhomosysteine; SAM, S-adenosylmethionine; IECs, intestinal epithelial cells; IEL, intestinal intraepithelial lymphocyte; DC dendritic cell; Th1, Th2, Th17, T-helper cells; NK-cell, natural killer T-cell; MC, mast cell; IL, interleukin; TNF, tumor necrosis factor; IFN, interferon; TLRs, Toll-like receptors; T-bet, T box transcription factor; RORγt, retinoid related orphan receptor γ; Tregs, Regulatory T-cells; FoxP3, Tregs forkhead box P3; GATA3, GATA Binding Protein 3; TGF-β, Transforming growth factor beta; NF-κB, nuclear factor κB; IgE, immunoglobulin E; FcεRI, FcepsilonRI; DAO, Diamine oxidase; HDC, L-histidine decarboxylase; PLC, phospholipase C; PIP2, phosphatidylinositol 4,5-diphosphate; DAG, diacylglycerol; IP3, inositol-1,4,5-triphosphate; PKC, protein kinase C; HNMT, Histamine-N-methyltransferase; NMH, N-methylhistamine; HR, histamine receptor; MAPK, mitogen-activated protein kinase pathway; NFAT, nuclear factor of activated T-cells; cAMP, cyclic adenosine monophosphate; CREB, response element binding protein; AP-1, activator protein 1 (original scheme).

As mentioned earlier, the activation of each HRs subtype stimulates various signaling pathways and diverse immunological activities. Given the lack of knowledge on the molecular interactions of histamine and intestinal HRs in IBD, we will attempt to map the main (and putative) intestinal immune responses and signaling pathways that may directly or indirectly influence the development of IBD (Figure 1).

According to the classical signaling outlined above, H1R signaling occurs in the gut via the activation of PLC to produce IP3 and DAG, which, in turn, triggers PKC protein activation and an increase in cytosolic Ca^2+^, followed by protein phosphorylation and transcription of NF-κB, NFAT, cAMP, CREB, and AP-1 [30]. Importantly, NF-κB is one of the main regulators of immunological processes in IBD, capable of regulating the expression of TNF-α, IL-1, IL-6, IL-12, and IL-23 [71]. Therefore, hypothetically, a number of components of the IBD pathway can be activated by elements of the H1R pathway (and other HRs, since they all terminate in NF-κB transcription) and vice versa. Accordingly, H1R and other HRs can work synergistically to activate the NLRP3 inflammasome, a member of the NOD-like receptor (NLR) family that is highly expressed in the gut and involved in the initiation of inflammation, by inducing NF-κB (and MAPK in the case of H4R). It further leads to the increased production of proinflammatory cytokines [72]. NLRP3 inflammasome controls the secretion of the proinflammatory cytokines IL-1β and IL-18. At the same time, NF-κB activation makes it possible to rapidly stimulate the expression of pro-IL-1β and pro-IL-18, which are then cleaved by Caspase-1 into mature IL-1β and IL-18.

H1R activates and induces Th1 responses [73]. At the same time, *H1R* knockout mice have an improved Th2 profile due to decreased Th1 responses. Histamine is known to promote Th1 polarization by H1R and enhance Th1 responses, providing additional anti-Th2 signaling through the increased production of IFN-γ, IL-2, and TNF-β [74]. It remains unclear precisely how H1R influences the polarization of T-cells. It has been established that Th1 and Th17 activity increases in CD, whereas Th2 activity rises in UC. Inflammation, cytotoxicity, and hypersensitivity reactions are all part of Th1-mediated cellular immunity. Therefore, the production of IL-12, IL-18, IL-27, and IL-23, which is already increased in CD, is necessary for H1R-mediated Th1 differentiation. In addition, H1R increases the release of IL-12, and IL-18 promotes neutrophil recruitment in colon tissue [75]. Intestinal DCs can be found proximate to degranulated mast cells, especially in mucosa. When activated, they can release proinflammatory IL-6, IL-12, and IL-18, thus regulating the responses of Th1 cells polarized via H1R [76]. Therefore, H1R has a potential proinflammatory effect which aggravates CD. Although IL-4 plays a crucial role in Th2 differentiation, anti-Th2 H1R activity may have a preventive effect on the development of UC. However, it is important to remember that the development of Th1 prevents the emergence of Th2 by increasing the production of IFN-γ and TNF-α, both of which are elevated in CD and UC. This suggests that H1R-mediated Th2 suppression will not have a significant protective effect against disease progression when used to influence the course of IBD. It should be noted that anti-Th2 activity of H1R is just another indirect way of influencing T-cell activity. In addition, it should be remembered that due to complex crossimmune interactions in the inflamed intestine, it is impossible to assess the unequivocal effect of one component on the development of a multifactorial disease. Further research is needed to investigate molecular immunopathological H1R-associated signaling and its possible synergistic effects on the components of IBD pathways.

In contrast to the H1R pathway, H2R signaling in the gut is mediated by the activation of the protein adenylyl cyclase, which increases the level of cyclic adenosine monophosphate and activates PKA [40]. Inflammatory and immunological responses are suppressed by the activation of H2R, which increases IL-10 production and inhibits IL-12, TNF-α, IL-23, and IL-27 [74]. It was revealed that H2R activates cAMP to inhibit the TLR-associated NF-κB and AP-1 pathways [65]. In addition, H2R suppresses Th1 and Th2 polarization, promoting polarization to Tregs [19]. Along with H1R, it is completely unknown how H2R modulates T-cell polarization. An anti-inflammatory role for H2R in relation to UC may be assumed given that Th2 activity is elevated in UC and that H2R inhibits these T-cells. Furthermore, Th17 will be suppressed in the presence of positive Tregs polarization and IL-10 production, thus reducing inflammation in CD [20]. We can, therefore, conclude that H2R exhibits anti-inflammatory effects in inflammatory diseases based on the results of the currently available research and our hypotheses. Further in-depth research on the anti-inflammatory potential of H2R is needed for the development of therapeutic approaches against complex immune-mediated IBD.

Despite the high degree of homology between H3R and H4R, there is currently no reliable evidence that H3R is present in human intestinal tissues. H4R in the gut blocks the H2R signaling cascade, which, in turn, prevents cAMP synthesis, Ca^2+^ accumulation, and MAPK activation [13]. H4R supports Th1 and Th2 polarization [19]. Since Th1 and Th2 are associated with a forceful proinflammatory response through the secretion of IFN-γ, TNF-β, IL-2, IL-4, IL-5, and IL-13, this may adversely affect the development of IBD and lead to significant intestinal damage [77]. Therefore, the polarization of Th1 and Th2 caused by H4R is expected to contribute to the worsening of IBD. In addition, there is an increase in neutrophil infiltration in the intestine and the production of proinflammatory IL-6, CXCL1, and CXCL2 via H4R, which worsens the IBD state [67]. It is critical that the promotion of H4R-associated neutrophil immunopathology in the gut allows for both damage to healthy intestinal tissue, which exacerbates the course of IBD, and protection against sepsis by limiting the penetration of bacteria into the damaged tissue. The final outcome is determined by a number of variables, including the severity of the disease, the recommended IBD therapy and any side effects, and the results of immune interactions. Furthermore, H4R has been suggested to stimulate Th2 cell development and negatively affect inflammation by stimulating NK-cells’ chemotaxis with histamine [21]. Thus, H4R exhibits a proinflammatory effect on inflammation. However, not enough studies have been conducted to date for a clear understanding of the immunopathologic and synergistic interactions of H4R in IBD. There appear to be significant differences in the potential effects of the molecular signaling pathways of different HRs on the onset of IBD. The uniqueness of HRs itself, the manipulations performed with the studied HR, and the complexity of the crossimmune interactions between IBD and HRs signaling cascades may all contribute to these differences. We hope that further research can answer the remaining questions and allow us to better understand this issue.

## 4. Antagonists and Agonists of HRs—Possible IBD Therapy?

To date, effective treatments for IBD include anti-TNF therapy, Janus kinase inhibitors, and antibodies that suppress leukocyte migration through the gastrointestinal tract. In addition, as a result of the study of various aspects of the disease etiology, new therapeutic drugs are actively developed and undergo clinical trials for the treatment of IBD [78]. Specifically, TLR9 agonist (cobitolimod), sphingosine 1-phosphate (S1P) receptor modulating agonists (estrasimod, ozanimod), and anti-IL-23 agents (mirikizumab) are under investigation. The possible therapy of IBD with HRs antagonists is also considered, which will be discussed below.

Following histamine detection at the beginning of the 20th century, further research was focused on the development of antihistamines—compounds that competitively block histamine receptors in the body, thereby inhibiting their multiple effects [79]. The creation of new ones and re-evaluation of existing ligands is ongoing due to the high risk of side effects of HRs chemical ligands as well as their specificity and inconsistent efficacy. The main focus of contemporary antihistamine research is on the search for different chemical ligands and the application of further experimental techniques. Finding suitable medications for complex conditions such as IBD is extremely difficult [80]. However, a number of HRs antagonists have been discovered to date that have therapeutic potential for IBD.

It is now well established from a variety of studies that H1R antagonists are among the most traditional medicines that work by preventing histamine from binding to H1R, and thereby reducing allergy symptoms. As already mentioned, H1R mainly causes proinflammatory responses in the intestine. However, the role of H1R in the pathogenesis of IBD is still unclear. Patients with IBD have been shown to benefit from the H1R antagonist loratadine, also known as ethyl 4-(8-chloro-5,6-dihydrobenzo[1,2]cyclohepta[2,4-b]pyridin-11-ylidene)piperidine-1-carboxylate) [81]. In particular, plasma histamine levels were not significantly reduced in IBD patients who received very low doses of loratadine as an adjunct to IBD treatment. Compared to UC, the effect was more pronounced with CD. Nevertheless, it is difficult to assess the clinical significance of the obtained results because histamine levels also decreased among IBD patients who did not receive loratadine. In view of the lack of knowledge of H1R in the context of IBD, further research is needed to clarify, in particular, the potential therapeutic effect of H1R antagonists on IBD.

In IBD, H2R has been shown to demonstrate anti-inflammatory effects. However, H2R antagonists have a completely different impact on the development of IBD. Thus, H2R antagonists are used to reduce gastric acidity, but they also alter the composition of the gut microbiota, thereby increasing the possibility of adverse IBD outcomes [17,18]. However, there is not enough evidence to support or disprove the true risks of an increase in IBD complications. More observational studies are needed to provide sufficient data to suggest a cumulative risk of IBD following the use of H2R antagonists. It is also important to consider the potential H2R partial/full agonists to ameliorate the putative beneficial effects in intestinal inflammation given the anti-inflammatory effect of H2R on the progression of IBD.

As previously discussed, H4R has an obvious proinflammatory activity in IBD, but the results of studies of several H4R antagonists point to a new pharmacological approach for the treatment of IBD. For example, the selective antagonist JNJ7777120 (1-[(5-chloro-1H-indol-2-yl)carbonyl]-4-methylpiperazine) is the first discovered highly selective H4 antihistamine compound [82]. It improves the clinical and histological symptoms of DSS colitis and attenuates the inflammatory cytokine response [67]. It inhibits histamine-induced chemotaxis and Ca^2+^ influx into mast cells. The antagonist JNJ7777120 significantly reduces macroscopic damage to the colon, attenuates the increase in tissue edema, inhibits the increase in myeloperoxidase levels and the influx of neutrophils, and decreases the level of TNF-α in the colon tissue. However, the exact cellular mechanisms underlying the anti-inflammatory effects in the gut are still not fully understood. It has also been reported that the effect of JNJ7777120 exposure is highly dependent on the specific species (mice, rats, etc.), which is important to consider in further studies [38]. Other selective H4R antagonists also reduced manifestations of chemically induced colitis. In particular, thioperamide (N-cyclohexyl-4-(3H-imidazol-4-yl)piperidine-1-carbothioamide) and JNJ10191584 ((6-chloro-1H-benzimidazol-2-yl)-(4-methylpiperazin-1-yl)methanone) reduced TNBS colitis [83,84]. These antagonists reduced macroscopic colon damage, neutrophil recruitment, myeloperoxidase levels, and inhibited cytokine release. The anti-inflammatory effect is due, at least in part, to the inhibition of aberrant TLRs signaling via DCs, resulting in the decreased production of TNF-α and IL-6 [85].

Thus, the discussed antagonists can be considered as a potential therapy for IBD. Regarding that, we have indicated mainly the compounds that demonstrate effectiveness in reducing gut damage and inflammation. It is also reasonable to speculate on possible H2R agonists as a treatment for IBD. Furthermore, it is worthy of note that the effects of HRs agonists and antagonists may differ significantly depending on the design of the study and the subject (human/animal models). Consequently, the obtained results shall not be interpreted unambiguously. The available evidence and contradictory data highlight the need for further research into the function of HRs subtypes and potential therapeutic potential in the gut in the context of IBD-associated inflammation. Of special interest is a possible model of histaminergic therapy for IBD implicating the combined use of antagonists and agonists, which is expected to achieve maximum benefits and avoid side effects. For example, the use of H4R antagonists simultaneously with an H2R agonist is a promising approach. However, taking into account histamine and HRs dependence on a number of intracellular and extracellular factors in the gut and their variability, complex antagonist/agonist treatment should be implemented with due circumspection.

In the context of existing therapeutic approaches, attention should also be paid to the possible negative consequences of the use of known therapies that may be directly or indirectly involved in the signaling pathways of histamine and its receptors.

Thus, anti-TNF therapy of IBD can affect mast cells’ TNF-α activity. Infliximab (IFX), an anti-TNF-α antibody, has been shown to inhibit the accumulation of mast cells in the colon, resulting in a decrease in TNF-α levels and attenuation of colon inflammation in an azoxymethane (AOM)/DSS mice model [86]. However, the intravenous administration of IFX can cause immediate infusion reactions, leading to a massive release of histamine [87]. Given that histamine currently demonstrates more proinflammatory rather than anti-inflammatory effects on the gut in IBD, the use of anti-TNF therapy (in particular, IFX) in patients with CD and UC may be accompanied by additional risks of inflammation. It has been reported that the intravenous administration of corticosteroids and antihistamines to patients treated with IFX resulted in a reduction in infusion reactions [88]. Thus, it might seem reasonable to use anti-TNF therapy in combination with corticosteroids/antihistamines not only to prevent serious adverse effects but also to reduce inflammation in IBD.

Administration of 5-Aminosalicylic Acid (5-ASK), a well-known anti-inflammatory drug, to 5-ASK sensitive patients has been shown to inhibit COX-1 enzyme activity, induce histamine release, and leukotriene synthesis [89]. Consequently, the treatment of IBD by 5-ASK may be associated with additional risks and, conversely, exacerbate the condition of patients with active disease.

## 5. Polymorphism of DAO and HNMT and Impact on IBD

The detection of gene polymorphism, in particular, single nucleotide polymorphism (SNP), enables to use them as specific markers in studies focused on the investigation of pathophysiology and genetics of a certain disease. Regarding that, some SNP can directly or indirectly affect the progression of existing disorders or the onset of new ones.

Taking into account the fact that IBD is, among other things, a genetically mediated disease and a number of loci are associated with IBD, the search and detection of possible relationships with SNP among genes that are directly associated with the histamine signaling pathway and HRs could be an additional approach to affect IBD progression. Thus, as a result of DAO and HNMT screening in homogenates of intestinal biopsy specimens from patients with IBD, four DAO SNP were detected: C995T (*Ser332Phe*), C47T (*Thr16Met*), C4106G (*His646Asp*), and G-4586T, as well as three HNMT SNP: C314T, A595G, and A939G, which resulted in protein amino acid substitutions or altered regulatory elements in the gene promoter [90,91,92]. It has been established that the detected SNP promotes a decrease in protein activity and stability, which, in turn, can impair histamine metabolism regulation in the inflamed gut. However, said studies were conducted on a small patient population, thus not allowing for the assessment of unambiguously possible associations of the detected SNP with IBD. Furthermore, another study in respect of HNMT and DAO SNP on a large subset of UC patients has shown them to have *Thr105Ile* and *His645Asp* SNP in HNMT and *His645Asp* in DAO [93]. As a result, conclusive evidence was provided suggesting the correlation of His645Asp SNP in DAO with UC severity. Mucosal DAO activity is known to be reduced in IBD, supporting the hypothesis that individuals with SNP in DAO may develop increased susceptibility to UC [94]. Additionally, the analysis of possible correlations between rs1049793 SNP in DAO and CD has shown it to be an inappropriate marker of clinical and endoscopic activity in CD [95]. The lack of association between SNP in HNMT and UC may indicate a relative contribution of HNMT to histamine metabolism in intestinal tissue, since HNMT activity therein is almost negligible compared to DAO activity. Nevertheless, HNMT gene polymorphism has been found to be associated with decreased enzymatic activity, leading to impaired histamine clearance [96]. Furthermore, no correlation of HNMT with UC has been detected in another study considering duodenal susceptibility to UC in a Chinese population with HNMT SNP *C314T* [97].

Thus, currently, there are very few studies focused on the search for possible associations of SNP in genes that are directly related to histamine signaling pathways. The confirmed correlation between DAO SNP and UC is an indirect marker for disease severity. Further identification of SNP associated with histamine pathway components in the gut may have significant consequences for our understanding of IBD progression and promote its early diagnosis.

## 6. Future Perspectives and Concluding Remarks

It is a matter of fact that histamine plays an important role in the digestive tract. Available data indicate its involvement in neuroimmune interactions sustaining normal gut functioning. In addition, histamine has also been found to mediate pathogenic conditions in the gut by directly or indirectly modulating immune responses.

The speculation on the role of histamine, HRs, and associated signaling pathways in the inflammatory response allows for considering this mediator as one of the key participants in the pathogenesis of IBD. In particular, H1R and H4R seem to be associated with the proinflammatory effect that exacerbates IBD progression, while H2R can have an anti-inflammatory effect in active disease. However, real molecular mechanisms underlying histamine/HRs-associated signaling pathways and responsible for inducing inflammation in IBD are still unclear. For example, there is a problem of HRs cross-signaling in the gut along with the lack of data relating to possible synergistic HRs effects on the components of IBD signaling pathways. In addition, there is currently insufficient evidence to form a clear concept on histamine/HRs signaling in the inflamed gut and its implications for IBD. The demonstrated proinflammatory and anti-inflammatory effects of different HRs in IBD require further studies.

Along with histamine, mast cells may have an important role regarding involvement in inflammation by mediating immune responses. Available studies on the role of mast cells in IBD have demonstrated a proinflammatory effect in the disease. However, there is still no clear understanding of the molecular mechanisms underlying mast cell functioning in IBD, due to the multifactorial crosstalk of the gut immune system pathways.

The search for possible SNP associations in genes related to histamine signaling pathways is crucial for revealing IBD onset/progression mechanisms. Said studies may have a significant role for diagnosis and clinical prognosis in patients with IBD.

It is hoped that further research in this field will ensure new insights into histamine contribution to the etiology and pathogenesis of IBD, thus providing the development of novel therapeutic approaches in IBD treatment, for example, based on agonists/antagonists of HRs or components of associated signaling pathways.

## Figures and Tables

**Table 1:** Sources of histamine.

Sources	Site of Production	References
Endogenous	Immune and nonimmune cells: endothelial cells, nerve cells, histaminergic neurons, intestinal epithelial cells (IECs), neutrophils, eosinophils, monocytes, macrophages, DCs, T- and B-cells, and Langerhans cells	[32,33]
Exogenous	Food: cheese, wine, sauerkraut, soy sauce, jerky, and seafood; Bacteria: *Escherichia coli*, *Lactobacillus vaginalis*, *Lactobacillus reuteri*, *Morganella morganii*, *Hafnia alvei*, *Proteus vulgaris*, *Proteus milabilis*, *Enterobacter aerogenes*, *Raoultella planticola*, *Raoultella ornithinolytica*, *Citrobacter freundii*, *Pseudomonas fluorescens*, and *Photobacterium damselae*	[10,11,34,35,36]

## Data Availability

No new data were created or analyzed in this study. Data sharing is not applicable to this article.

## Abbreviations

IBD	Inflammatory bowel disease
CD	Crohn’s disease
UC	Ulcerative colitis
GI	Gastrointestinal tract
DAO	Diamine oxidase
HDC	L-histidine decarboxylase
FcεRI	FcepsilonRI
SP	Substance P
IgE	Immunoglobulin E
PLC	Phospholipase C
PIP2	Phosphatidylinositol 4,5-diphosphate
DAG	Diacylglycerol
IP3	Inositol-1,4,5-triphosphate
PKC	Protein kinase C
GM-CSF	Granulocyte-macrophage colony-stimulating factor
SCF	Stem cell factor
HNMT	Histamine-N-methyltransferase
SAM	S-adenosyl-L-methionine
NMH	N-methylhistamine
HIT	Histamine intolerance
HRs	Histamine receptors
GPCR	Protein-coupled receptor
moDCs	Monocyte-derived dendritic cells
MAPK	Mitogen-activated protein kinase pathway
FoxP3	Tregs forkhead box P3
VH	Visceral hypersensitivity
Ox	Oxazolone
AOM	Azoxymethane
5-ASK	5-Aminosalicylic Acid
IFX	Infliximab
MPO	Myeloperoxidase
NLR	NOD-like receptor
SNP	Single nucleotide polymorphism
IECs	Intestinal epithelial cells
IELs	Intestinal intraepithelial lymphocytes
DCs	Dendritic cells
Th	T-helper cells
Tregs	Regulatory T-cells
NK-cells	Natural killer T-cells
IL	Interleukin
TNF	Tumor necrosis factor
IFN	Interferon
TLRs	Toll-like receptors
NFAT	Nuclear factor of activated T-cells
cAMP	Cyclic adenosine monophosphate
CREB	Response element binding protein
AP-1	Activator protein 1
DSS	Dextran sulfate sodium
PAMPs	Pathogen-associated molecular patterns
NF-κB	Nuclear factor κB
PKB	Protein kinase B
PAR-2	Protease-activated receptor-2
mTOR	Mammalian target of rapamycin
APCs	Antigen-presenting cells
TNBS	2,4,6-trinitrobenzenesulfonic acid
